# Circulating sterols as predictors of early allograft dysfunction and clinical outcome in patients undergoing liver transplantation

**DOI:** 10.1007/s11306-016-1129-z

**Published:** 2016-10-24

**Authors:** Uta Ceglarek, Kathleen Kresse, Susen Becker, Georg Martin Fiedler, Joachim Thiery, Markus Quante, Robert Wieland, Michael Bartels, Gabriela Aust

**Affiliations:** 1 Institute of Laboratory Medicine, Clinical Chemistry and Molecular Diagnostics, University Hospital Leipzig, University of Leipzig, 04103 Leipzig, Germany; 2 LIFE–Leipzig Research Center for Civilization Diseases, University Leipzig, Philipp-Rosenthal-Strasse 27, 04103 Leipzig, Germany; 3 Research Laboratories and Clinic of Visceral, Transplantation, Thoracic, and Vascular Surgery, University Hospital Leipzig, University of Leipzig, Liebigstraße 19, 04103 Leipzig, Germany; 4 Department of Laboratory Medicine, Institute of Clinical Chemistry, Inselspital, Bern University Hospital, University of Bern, Bern, Switzerland

**Keywords:** Liver transplantation, Sterol metabolism, MEAF

## Abstract

**Introduction:**

Sensitive and specific assessment of the hepatic graft metabolism after liver transplantation (LTX) is essential for early detection of postoperative dysfunction implying the need for consecutive therapeutic interventions.

**Objectives:**

Here, we assessed circulating liver metabolites of the cholesterol pathway, amino acids and acylcarnitines and evaluated their predictive value on early allograft dysfunction (EAD) and clinical outcome in the context of LTX.

**Methods:**

The metabolites were quantified in the plasma of 40 liver graft recipients one day pre- and 10 days post-LTX by liquid chromatography/tandem mass spectrometry (LC–MS/MS). Plant sterols as well as cholesterol and its precursors were determined in the free and esterified form; lanosterol in the free form only. Metabolites and esterification ratios were compared to the model for early allograft function scoring (MEAF) which is calculated at day 3 post-LTX from routine parameters defining EAD.

**Results:**

The hepatic esterification ratio of all sterols, but not amino acids and acylcarnitine concentrations, showed substantial metabolic disturbances post-LTX and correlated to the MEAF. In ROC analysis, the low esterification ratio of β-sitosterol and stigmasterol from day 1 and of the other sterols from day 3 were predictive for a high MEAF, i.e. EAD. Additionally, the ratio of esterified β-sitosterol and free lanosterol were predictive for all days and the esterification ratio of the other sterols at day 3 or 4 post-LTX for 3-month mortality.

**Conclusion:**

Low ratios of circulating esterified sterols are associated with a high risk of EAD and impaired clinical outcome in the early postoperative phase following LTX.

**Electronic supplementary material:**

The online version of this article (doi:10.1007/s11306-016-1129-z) contains supplementary material, which is available to authorized users.

## Introduction

Liver transplantation (LTX) is a life-saving treatment option for patients suffering from a variety of liver diseases including acute and chronic liver failure, hepatocellular carcinoma and fulminant hepatitis. Over the past 50 years, significant improvements in immunosuppression, surgical technique and postoperative management of liver allograft recipients have paved the way for the clinical success of LTX which has finally become a standard therapy and clinical routine.

Primary graft dysfunction after LTX is a critical event and may display a broad clinical range from reversible graft dysfunction, known as early allograft dysfunction (EAD), up to an irreversible dysfunctional state called primary non function which represents an indication for urgent re-transplantation (Uemura et al. 2007). EAD has been shown to have negative impact on both, patient and graft outcome. Early and accurate assessment of graft function following LTX is critical for timely therapeutic intervention resulting in reduced mortality and reduced numbers of re-transplantation. Moreover, sensitive and specific assessment of the hepatic metabolism after LTX remains essential for early detection of postoperative dysfunction and consecutive adjustment of the therapeutic regime. In clinical routine, liver cell function is assessed by the activity of in part specific enzymes such as alanine and aspartate aminotransferase (ALT, AST), bilirubin and parameters of the coagulation system such as the international normalized ratio of prothrombin (INR) to define EAD (Olthoff et al. 2010; Lee et al. 2014). Recently, a model for the quantitative assessment of EAD after LTX has been validated (model for early allograft function scoring, MEAF) (Pareja et al. 2015). This score, based on the parameters ALT, bilirubin and INR calculated at day 3 post-LTX, provides improved graft function assessment than the single parameters (Pareja et al. 2015) while at the same time being a predictor of both recipient and graft survival. However, all single parameters used to calculate the MEAF might be influenced by the ischemic period during organ preservation, liver graft quality, coagulation factor substitutions and blood or plasma transfusions, as well as nutrition. Thus, evaluation of the actual parenchymal function and precise distinction between a toxic and hypoxic liver injury can be challenging (Hickman et al. 1997; Shaked et al. 1997; Tanaka et al. 2000; Sear 2002) since routine parameters may rather reflect pre- and perioperative liver cell injury due to ischemia and organ preservation than the residual metabolic capacity of the transplanted liver.

To characterize new postoperative parameters that might assess more adequately or even earlier EAD compared to the MEAF, we here examined various liver specific metabolic pathways including cholesterol synthesis and secretion, sterol esterification, branched-chain and aromatic amino acids and fatty acid beta-oxidation by liquid chromatography/tandem mass spectrometry (LC–MS/MS). Free and esterified sterols, amino acids and acylcarnitines were determined together with the routine parameters by high throughput methods 1 day pre- and 10 days post-LTX and related to clinical outcome and the MEAF.

The change of sterol concentrations before and after LTX was studied in patients with end-stage primary biliary cirrhosis and acute liver failure (Nikkilä et al. 1992, 2005, 2008). Here, we focused on sterol esterification, which depends strongly on liver function and was already discussed as a marker for it many years ago (Jones et al. 1971; Simon et al. 1974). However, sterol esterification has not been studied in liver function after transplantation. Conversion of free sterols into sterol esters is catalyzed by the lecithin cholesterol acyltransferase (LCAT). This enzyme is synthesized in the liver and its activity is a sensitive parameter of liver disease (Borowsky et al. 1980; Breier et al. 1983). Using our LC–MS/MS approach, we are able to determine sterol esterification by simultaneous quantification of the free and esterified sterol including cholesterol, endogenous and non-endogenous sterols.

## Materials and methods

### Subjects

A total of 40 Caucasian men (n = 29) and women (n = 11) undergoing LTX were consecutively recruited from July 2008 to July 2009. Table 1 summarizes baseline and hospitalization characteristics of patients pre-LTX. 27 out of the 40 patients (27/40) had alcoholic liver disease (ALD) as baseline liver disease. 20/27 of these patients were men. Alcohol abuse is a mostly problem of men: two-thirds of people with alcohol use disorders are men (Deleuran et al. 2015). We did not record the personalized diet of each patient. The patients were fed according to the following suggestions: (i) patients without any complication: post-LTX oral light diet, after 2–3 days stepwise increasing caloric food; (ii) patients which could not be fed orally within the first 1–4 days post-LTX but afterwards: Ringer‘s solution i.v., after 4 days oral light diet and increasing caloric food; patients with complications and probably no oral nutrition after 4–5 days will be possible: enteral nutrition according to the patients demand.

**Table 1:** Baseline and hospitalization characteristics of the patients pre-LTX

Parameter (unit)	Number (%)	Mean ± SD or median (interquartile range)
Recipient information		
Number of patients	40	
Age (years)		53.68 ± 8.35
Gender (male)	29 (72.5)	
Baseline liver disease		
Alcoholic liver disease (ALD)	27 (67.5)	
Hepatitis B/C	4 (10.0)	
Hemochromatosis	2 (5.0)	
Acute liver failure (ALF)	3 (7.5)	
Posttransplant-liver complication	2 (5.0)	
Biliar disease	2 (5.0)	
Tumor (HCC)	13 (32.5)	
MELD		19.50 (12.75–35.25)
MEAF		4.72 (3.25–6.11)
Transplantation before study transplantation	3 (7.3)	
Mortality after 10 days	0 (0)	
Mortality after 30 days	1 (2.5)	
Mortality after 3 months	3 (7.5)	
Organ failure after 30 days	2 (5.0)	
Organ failure after 3 months	5 (12.5)	
Organ failure after 18 months	8 (20.0)	
Length of hospital stay (days)		30.50 (21–39.25)
Length of ICU stay (days)		13.00 (8.00–20.00)
Donor information		
Age (years) donor		54.58 ± 17.59
Gender (male) donor	22 (55.0)	
Gender mismatch		
No	25 (62.5)	
Male (D) → female (R)	4 (10.0)	
Female(D) → male (R)	11 (27.5)	
BMI (kg/m^2^)		26.18 (23.38–29.39)
Surgical information		
CIT (min)		597 ± 146
Arterial anastomosis delay (min)		60.000 (50.00–60.00)

Mean and standard deviation (SD), for non-normally distributed parameters median and interquartile ranges are given

*BMI* body mass index, *CIT* cold ischemic time, *D* donor, *ICU* intermediate care unit, *HCC* hepatocellular carcinoma, *MEAF* model for early allograft function scoring, *MELD* model for end-stage liver disease, *R* recipient

Three patients had to undergo re-transplantation after their first LTX. Here, only the data after re-transplantation were included. Two out of these three patients underwent a LTX 2 and 6 months before the second LTX because of thrombosis of the liver artery. In both cases blood was not collected after the first LTX. After the second LTX the determined parameters of both patients were between the 25th and 75th percentile of all patients. The third patient received the second LTX 4 days after the first LTX because of acute liver failure. This is one of the patients who died within 3 months after LTX.

### Blood samples

1–24 h pre-LTX (day 0) and each day post-LTX until day 10 (days 1–10) peripheral venous blood was collected. 80.0 % of all blood samples were collected between 4 and 7 am. and 9.1 % between 7 and 10 am. Because all patients were in the intensive care unit (ICU) post-LTX, taking blood samples depended on the treatment needs. Thus, the remaining 10.9 % of blood samples could not have been taken within the time frame 4–10 am. The blood was centrifuged immediately at 2500×*g* for 20 min to obtain EDTA-plasma. Samples were stored at −80 °C and thawed only once. To generate dried blood specimen, EDTA whole blood was dropped on filter paper (grade 903; GE Healthcare, Munich, Germany) dried at room temperature for 3 h and afterward stored in foil-barrier ziploc bags and desiccant packets (Whatman GmbH, Dassel, Germany) at –80 °C till analysis.

### Scores

The model used for end-stage liver disease (MELD) score was the crude MELD score. It was calculated with the following formula after recruitment of the patients (Kamath and Kim 2007): MELD = [0.957 ln(creatinine) + 0.378 ln(bilirubin) + 1.120 ln(INR) + 0.643] × 10 (United Network for Organ Sharing, UNOS; MELD Calculator; accessed March 30, 2015). MEAF was calculated with the following formula (Pareja et al. 2015) with max3POD = maximum variable values during the first 3 postoperative days: MEAF = score ALT_max3POD_ + score INR_max3POD_ + score bilirubin_3POD_. The MEAF could not be determined for two patients. Clinical outcome as 3-month mortality (n = 3 patients), 3-month mortality/organ failure (n = 5 patients), 12- and 18-month mortality (n = 5 patients) were collected retrospectively.

### Chemical and reagents

Methanol and isopropanol were purchased from Merck (Darmstadt, Germany) and acetyl chloride (p.a.) from Sigma-Aldrich (Steinheim, Germany). Water (HPLC grade) was obtained from J. T. Baker (Deventer, Netherlands). AA and AC reference isotope labeled standard kits (NSK A, NSK B; Cambridge Isotope Laboratories, Andover, USA) were used as internal standard. 3*N* butanolic HCl was made in-house using butanol (spectroscopy grade, Merck Darmstadt, Germany). Dried blood controls for amino acids and acylcarnitines (Level 1 and Level 2) were obtained from Chromsystems (Munich, Germany).

### Clinical laboratory characteristics

All analyzed parameters are summarized in Supplemental Table 1. ALT and AST, gamma glutamyl transferase (GGT), glutamate dehydrogenase (GLDH), creatinine and bilirubin serum concentrations were analyzed using Cobas 6000 and 8000 analyzers (Roche, Mannheim, Germany). The prothrombin assay was performed in citrate plasma to determine the INR using an ACL TOP 700 System (Instrumentation Laboratory, Lexington, USA).

### LC–MS/MS analysis

Sterol analysis included the free and esterified plant sterols brassicasterol (BR), campesterol (CA), β-sitosterol (SI) and stigmasterol (ST), the cholesterol biosynthesis precursors lanosterol (LA) as well as the sum parameter including desmosterol, zymosterol, 7-dehydrocholesterol (DEZY7DHC), and cholesterol itself (CH). They were analyzed by LC–MS/MS (Lembcke et al. 2005; Becker et al. 2015). 25 µl of the supernatants were injected onto the analytical column (Chromolith SpeedROD RP-18e, 50 × 4.6 mm, monolithic column, Merck KGaA, Darmstadt, Germany). An API 4000 triple quadrupole mass spectrometer with an atmospheric pressure photoionization source (AB Sciex, Darmstadt, Germany) was used. The quantification of free and esterified sterols was performed according to ISO DIN 17025 and ISO DIN 15189 including an external 4-point calibration with three quality controls at different concentrations. Data were acquired using Analyst software (version 1.5.1). The sterol esterification ratio was calculated with the following formula: esterified sterol/(free sterol + esterified sterol) × 100.

Analysis of amino acids and acylcarnitines was performed according to published protocols (Ceglarek et al. 2002; Brauer et al. 2011). Briefly, 3 mm dried blood spots were extracted with 100 µl of the internal standard solution and centrifugated at 3000×*g* for 10 min at room temperature. After butanolic esterification, samples were analyzed by liquid chromatography tandem mass spectrometry (API 2000, Sciex, Darmstadt, Germany).

### Statistical analyzes

Before statistical analysis, non-normally distributed parameters were logarithmically transformed to approximate normal distribution. Mean and standard deviation (SD), for non-normally distributed parameters median and the interquartile range (25th–75th percentile) were used. All statistical computations were performed using SPSS version 20.0 (IBM, Ehningen, Germany). *p* values less than 5 % were considered as significant.

Receiver Operating Characteristic (ROC) analyzes were used to assess the diagnostic power of the new parameters for discrimination between patients. The observed area under the ROC curve (AUC) was tested against the null-hypothesis (AUC = 0.5). Optimal cut-offs for the evaluated parameters were derived from the ROC curves by maximizing the sum of sensitivity and specificity (Youden-Index).

## Results

Clinical and anthropometric characteristics of patients are summarized in Table 1. The study group included 40 LTX patients with a mean age of 53.7 ± 1.3 (35–69) years. Alcoholic liver disease (ALD) was the predominant underlying liver disease, followed by hepatitis B/C.

### The esterification ratio of sterols showed substantial disturbances pre- and post-LTX

**Fig. 1:**
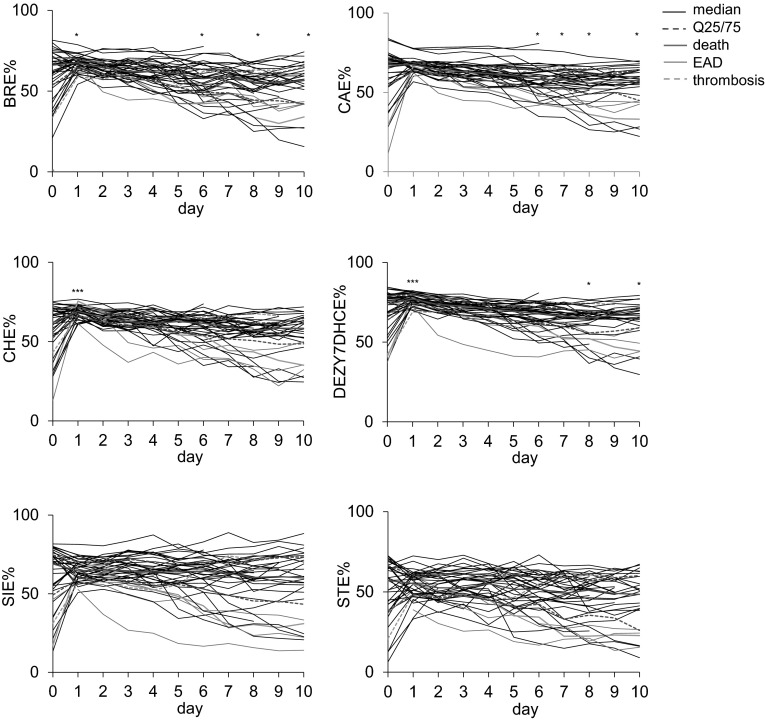
Time course of the ratio of esterified sterols The median and the 25 and 75 % interquartile ranges (Q25/75) are shown in *red*. The patients who died within 3 months (n = 3) and the patients with organ failure (n = 2) are shown in *pink* and in *green*, respectively. The *continuous green line* represents a patient with EAD and high MEAF whereas the *green broken line* indicates a patient who lost the liver 2 months post-LTX due to thrombosis of the hepatic artery. The differences between day 0 pre-LTX to each day post-LTX are indicated (Wilcoxon signed-rank test; ∗p < 0.05, ∗∗p < 0.01, ∗∗∗p < 0.001)

Substantial metabolic disturbances occurred immediately after LTX as seen in the time course of the esterification ratio of all sterols (Fig. 1) and of routine parameters, suitable to follow-up LTX, as ALT, AST, bilirubin, GGT, glutamate dehydrogenase (GLDH), and prothrombin time (PT). No or only low disturbances post-LTX were seen in circulating amino acids as well as acylcarnitines (data not shown).

The ratio of esterified sterols prior to LTX varied considerably for each sterol among the patients (Fig. 1); e.g. of CA between 11.8 and 83.9 %. Immediately post-LTX the esterification ratio of all sterols increased especially in patients with a low esterification ratio pre-LTX. The esterification ratio of all sterols post-LTX did not correlate to the cold ischemic time of the donor liver (data not shown). At day 1 the ratio of each esterified sterol, apart from ST, was higher than 50 % in all patients. From day 2 post-LTX the ratio of esterified sterols decreased continuously in at least half of the patients. Thus, we included the difference in the ratio of esterified sterols between day one to each other day after LTX into further analysis. In part, the difference in the esterification ratio between day 1 and 2 post-LTX was even more significant. At day 10, the ratio of esterified sterols varied among the patients partly as pre-LTX. For BR, CA and DEZY7DHC the median esterification ratio was even higher pre-LTX compared to that of day 10 post-LTX.

We found no significant differences between men and women concerning age and all routine and sterol data. Additionally, we found no differences between the 25 % youngest and 25 % oldest women. Thus, it is likely that the hormonal status/menopause had no effect on the results.

### The esterification ratio of sterols correlates inversely to the MEAF

**Fig. 2:**
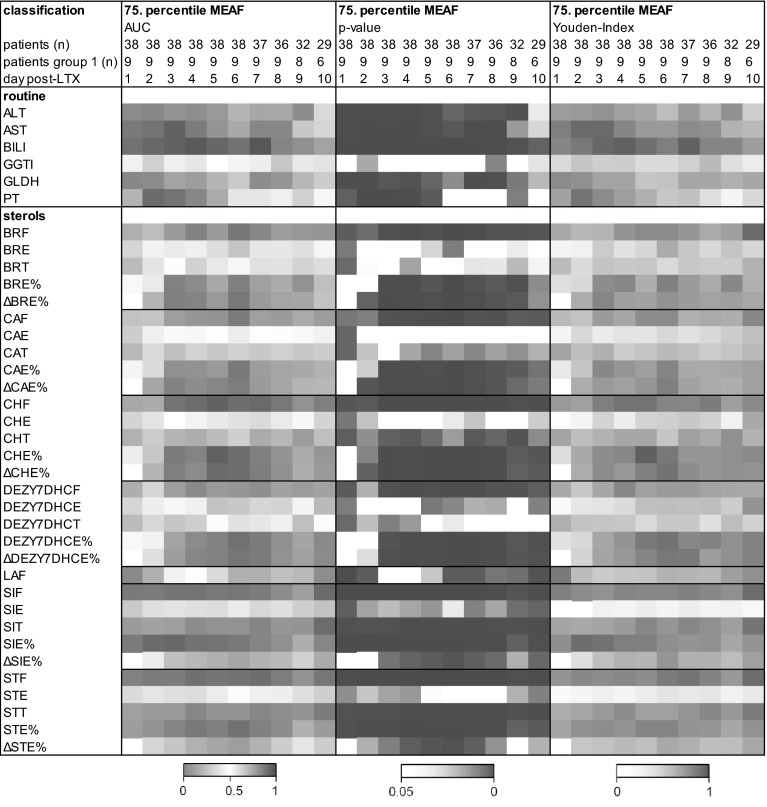
Sterols as predictors for a high MEAF receiver operating characteristic (ROC) analysis to verify which parameter predicts a high MEAF. The 75 % percentile of the MEAF was used as binary classifier, i.e. the 25 % of the patients with a MEAF ≥ 6.10 were compared to the others. Values for the area under the curve (AUC; 0–1), *p* values (<0.05–0) and the Youden-Index (0–1) are shown in heat maps. The Youden-Index, defining the maximum potential effectiveness of the parameter, was calculated from the AUC curves

Recently, the MEAF was identified to be most suitable for modeling EAD in patients undergoing LTX (Pareja et al. 2015). The score includes ALT, bilirubin and INR. Consequently, these parameters correlated to the MEAF (data not shown). Interestingly, the esterification ratio of the sterols correlated inversely to the MEAF almost every day except day 1, i.e. the higher the MEAF the lower the esterification ratio. The amount of free sterols and the MEAF correlated slightly. Supplemental Fig. S1a shows exemplarily that the data for free (SIF), esterified (SIE), total (SIT), and the ratio of esterified SI (SIE %) correlated to ALT, bilirubin, INR and MEAF whereas figure S1b illustrates the correlation between the esterification ratio of each sterol to the MEAF at days 3, 6, and 9 post-LTX. Finally receiver operating characteristic (ROC) analysis was performed. As binary classifier the 75 % percentile of the MEAF, e.g. the 25 % of the patients with a MEAF ≥ 6.10 were compared to the others. The low esterification ratio from day 1 of SI and ST and from day 3 of all the other sterols was predictive for a high MEAF (Fig. 2). Thus, high ratios of circulating esterified sterols probably predict a low risk of EAD. The Youden-Index, defining the maximum potential effectiveness and the cut-off of the esterification ratio, was calculated (Fig. 2). The highest Youden-Index was 0.82 for SIE % at day 2 post-LTX (cut-off 59.10 %) and 0.90 for CHE % at day 5 post-LTX (cut-off 57.80 %).

The other non-routine parameters such as amino acids and acylcarnitines either did not correlate or randomly correlated to the MEAF and are thus not predictive (Fig. S2).

### The ratio of esterified sterols predicts clinical outcome

**Fig. 3:**
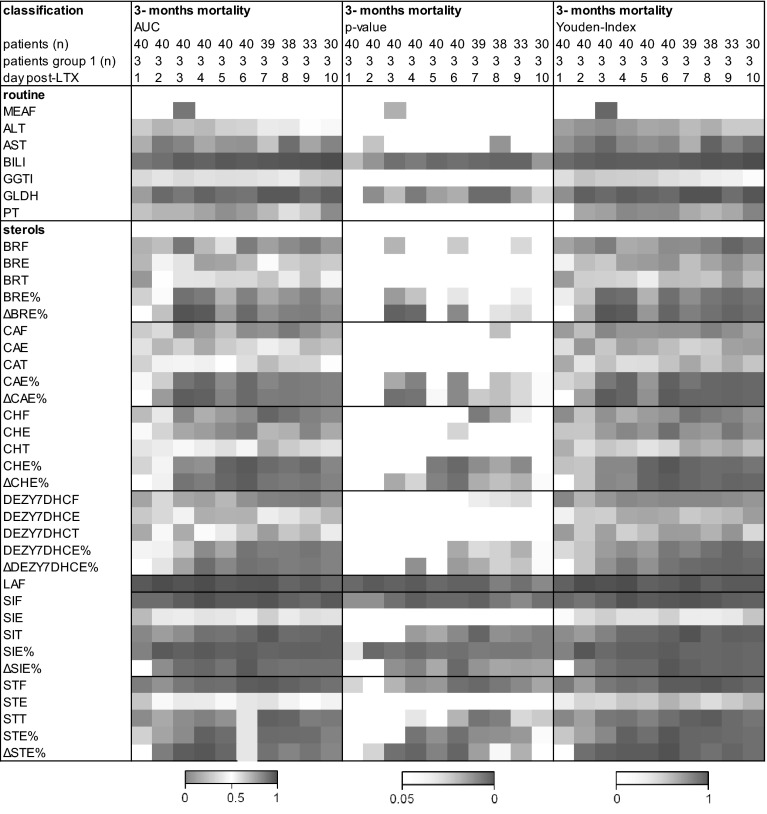
Sterols as predictors for 3 month mortality Receiver operating characteristic (ROC) analyzes to verify which parameter predicts clinical outcome. 3 month mortality was used as binary classifier. Explanations see Fig. 2

Finally, we evaluated the prognostic value of the assessed metabolic parameters on clinical outcome. Patients who died within the first three months after LTX very often showed esterification ratios of the sterols below the median of all patients or even the lowest values on many days (Fig. 1). The two patients who lost the organ had different profiles: the one with EAD was comparable to patients who died within 3 months whereas the patient who lost the liver 2 months post-LTX due to thrombosis of the hepatic artery with no signs of EAD and a low MEAF always had high esterification ratios of the sterols (Fig. 1). In the ROC analysis 3, 12, and 18 month mortality were used as binary classifiers. In Fig. 3, the AUC and the level of significance for 3-month mortality are given for each day. Among the routine parameters, bilirubin and GLDH were predictive on nearly all days as was the MEAF determined at day 3. Among the newly evaluated parameters, the sterols were especially interesting. Free LA (LAF), free ST (STF) and free and the ratio of esterified SI (SIF, SIE %) were predictive on all days post-LTX. The ratio of esterified as well as difference in the ratio of esterified BR (BRE, ΔBRE %) and CA (CAE, ΔCAE %) were predictive from day 3 post-LTX. For 12, and 18 month mortality the same results tended to be seen as for 3-month mortality, albeit at a lower or no level of significance. Finally, the Youden-Index was calculated (Fig. 3). The highest Youden-Index was 0.95 for SIE % (cut-off 57.52 %) and 1.00 for LAF at day 2 post-LTX (cut-off 0.27 mg/l) which are better than that of the MEAF calculated at day 3 post-LTX.

Circulating amino acids were not informative (Fig. S3). In tendency, several short- and medium-chain acylcarnitines showed predictive AUC levels, but they never or only on single days reached the level of significance.

## Discussion

Here, we identified a significant predictive value for the esterification ratio of circulating sterols on EAD and 3-month mortality in patients undergoing LTX.

Circulating sterols are composed of cholesterol, an essential component of membranes by regulating the fluidity of the bilayer, several precursors of cholesterol as lanosterol which are markers for cholesterol synthesis, and plant sterols which are exclusively derived from dietary sources (Kuksis 2001). Intracellular esterification and the subsequent packing into cytoplasmic lipid droplets detoxifies and sequesters sterols until they are required. The hydrophobic sterols are transported in circulation via lipoproteins to the peripheral cells (Huang et al. 2015).

The esterification ratio of sterols depends on two enzymes. Sterol esterification for synthesis of chylomicrons and very low-density lipoproteins (VLDLs) to transport synthesized sterols from the liver in the circulation is catalyzed by acyl-coenzyme A: cholesterol acyltransferase 2 (ACAT2) (Rogers et al. 2015). The LCAT, secreted by the liver into the blood, influences sterol esterification in circulating lipoproteins (Glomset and Verdery 1977; Dobiasova and Frohlich 1999; Jonas 2000). LCAT activity itself is a sensitive parameter for the severity of the liver disease (Borowsky et al. 1980; Breier et al. 1983). Thus, the esterification ratio of circulating sterols correlates to the number of intact hepatocytes. Overall, the plasma concentration of LCAT in normal liver function showed only minor variation (Kunnen and Van 2012) and varied only slightly in adult humans with age, gender, and smoking (Albers et al. 1982).

In our study, the ratio of each esterified sterol was significantly different between the patients pre-LTX with variances around 50–70 %. Of note was the marked increase in the esterification ratio immediately post-LTX for all sterols in patients that had shown low esterification ratios pre-LTX. From day two post-LTX the esterification ratio of all sterols started decreasing in more than 50 % of the patients. This uniform time course for all sterols suggests either that during LTX highly esterified sterols, typical for healthy individuals (Temel et al. 2003) have been transfused or that the LCAT activity of the donor liver is not disturbed immediately after LTX. Consistently, the ratio of all esterified sterols correlated strongly to routine parameters defining EAD and variables of the MEAF (Olthoff et al. 2010; Lee et al. 2014; Pareja et al. 2015). The low esterification ratio of SI and ST from day 1 and of the other sterols from day 3 was predictive to be among the 25 % of the patients with the highest MEAF. Although based on a small study cohort, these data suggest that the low esterification ratio of sterols is predictive for EAD.

Circulating plant sterols such as BR, CA, SI and ST solely result from dietary intake (Gylling et al. 2014; Ikeda 2015) and cannot be metabolized in the human organism except by being esterified predominantly in the liver (Chang et al. 2006). In our study, the esterification ratio of SI from day 1 and of BR and CA from day 3 as well as the difference in the esterification ratio day 1–2 of ST predicted a 3-month mortality after LTX. Interestingly, the free form of SI and ST was also predictive for a 3-month mortality from day 1 until day 10 post-LTX. The same association could be found for free LA. LA is a non-CH sterols that are active precursors in the cellular CH biosynthesis. Their CH-normalized concentrations are surrogate markers reflecting endogenous de novo synthesis of CH in the liver (Miettinen et al. 1990). The fact that free LA, SI, ST also predict 3-month mortality suggests that disturbances in hepatic cholesterol synthesis and cholesterol efflux by biliary excretion impact cholesterol homeostasis.

In addition, we also evaluated whether systemic levels of circulating amino acids were predictive for EAD and clinical outcome post-LTX. Altered amino acid metabolism is a hallmark of liver disease. Acute liver failure (ALF) is characterized by elevated levels of the circulating aromatic amino acids (AAAs) Phe, Trp and Tyr as well as Met. In contrast, reports on the concentrations of circulating branched-chain amino acids (BCAAs) Leu/Iso and Val have been conflicting (Blonde-Cynober et al. 1999; Honda et al. 2004). In chronic liver failure (CLF), decreased circulating BCAA and slightly elevated AAAs concentration have been reported consistently (Fischer et al. 1976; Watanabe et al. 1982). In the present study, the circulating level of all amino acids and the Fisher ratio, i.e. the ratio of the BCAAs to AAAs, did not correlate to the MEAF. Of note was the prediction of mortality for some amino acids and of organ failure only on day 1 after LTX which might be caused by blood transfusion or nutritional effects linked to caloric substitution. Overall, the patients with an unfavorable time course post-LTX received a different nutritional regimen compared to the patients with an uncomplicated healing. Probably the different scheme in nutrition explains the missing correlation between liver function and plasma amino acids and acylcarnitines. However, sterol esterification is not influenced by nutrition.

Finally, we also examined the carnitine shuttle and beta-oxidation of fatty acids. l-carnitine transports fatty acids into the mitochondria for subsequent β-oxidation, a process which results in its esterification to form acylcarnitine derivatives. As such, the endogenous carnitine pool is comprised of l-carnitine and various short-, medium- and long-chain acylcarnitines (McCoin et al. 2015). Thus, acylcarnitines play a decisive role in free fatty acid (FFA) oxidation, responsible for the transportation of acyl-coenzyme A into mitochondria, especially in liver and muscle cells. Acute liver failure with microvesicular steatosis is the consequence of severe impairment of mitochondrial beta-oxidation (Pessayre et al. 1999; Jaeschke et al. 2002). However, we did not find any correlation between l-carnitine/acyl carnitines and any of the routine parameters defining EAD and the MEAF. Long-chain acyl carnitines are sensitive biomarkers of acetaminophen (APAP)-induced hepatotoxicity in mouse models and human children (Bhattacharyya et al. 2013, 2014). High doses of APAP, the most widely used drug for the treatment of pain and fever, is the major cause of acute liver failure (ALF). In our study, none of the patients with acute liver failure had abused. Long-chain acylcarnitines accumulated, whereas free carnitine, medium and short-chain acylcarnitines decreased with the severity of the non-malignant chronic liver diseases, accompanied with corresponding alterations in enzyme activities of carnitine palmitoyl transferase 2 (CPT2) (Zhou et al. 2012).

In summary, we present promising data showing that sterols and especially their esterification ratios are suitable markers to follow up LTX and to predict EAD and clinical outcome. Our results clearly indicate the diagnostic potential of circulating sterols in the context of liver function assessment and justify further in-depth exploration.

## Electronic supplementary material

Below is the link to the electronic supplementary material.

Abbreviations of the determined parameters are given in Table S1. Each sterol was abbreviated with two letters (e. g. β-sitosterol: SI). The free, esterified and total concentration, the esterification ratio and the difference in the esterification ratio are given as SIF, SIE, SIT, SIE and ΔSIE %, respectively. Supplementary material 1 (DOC 142 kb)

Supplementary material 2 (PDF 361 kb)

Supplementary material 3 (PDF 15 kb)

Supplementary material 4 (PDF 15 kb)

## Abbreviations

ALD: Alcoholic liver disease
AUC: Area under the curve
EAD: Early allograft dysfunction
LC–MS/MS: Liquid chromatography–mass tandem spectrometry
LCAT: Lecithincholesterol acyltransferase
LTX: Liver transplantation
MEAF: Model for early allograft function scoring,
MELD: Model for end-stage liver disease
