# Supplementary material for: Circulating sterols as predictors of early allograft dysfunction and clinical outcome in patients undergoing liver transplantation
Source: Metabolomics. 2016 Oct 24;12(12):182. doi: 10.1007/s11306-016-1129-z (PMC5078158; doi:10.1007/s11306-016-1129-z)
Supplement: Supplementary file 1 — Abbreviations of the determined parameters are given in Table S1. Each sterol was abbreviated with two letters (e. g. β-sitosterol: SI). The free, esterified and total concentration, the esterification ratio and the difference in the esterification ratio are given as SIF, SIE, SIT, SIE and ΔSIE %, respectively. Supplementary material 1 (DOC 142 kb) [file 11306_2016_1129_MOESM1_ESM.doc]

**Supplemental Material**

**Circulating sterols as predictors of early allograft dysfunction and clinical outcome in patients undergoing liver transplantation**

**Table S1** Parameters determined

| **group/parameter** | **abbreviation** | **Unit** |
| --- | --- | --- |
| **coagulation** |  |  |
| international normalized ratio of prothrombin | INR |  |
| prothrombin time | PT | % |
| **liver** |  |  |
| model for early allograft function scoring | MEAF |  |
| alanine aminotransferase | ALT | µkat/l |
| alkaline phosphatase | API | µkat/l |
| aspartate aminotransferase | AST | µkat/l |
| conjugated bilirubin | BILD | µmol/l |
| unconjugated bilirubin | BILI | µmol/l |
| total bilirubin | BILIN | µmol/l |
| cholinesterase | CE | µkat/l |
| gamma glutamyl transferase | GGTI | µkat/l |
| glutamate dehydrogenase | GLDH | µkat/l |
| **proteins** |  |  |
| albumin | ALB | g/l |
| Total protein | PROT | g/L |
| **renal parameter** |  |  |
| creatinine | CRE | µmol/l |
| modification of diet in renal disease (GFR) | MDRD | ml/min/1.73m² |
| **sterols** |  |  |
| brassicasterol free | BRF | mg/l |
| brassicasterol ester | BRE | mg/l |
| brassicasterol total | BRT | mg/l |
| esterification rate of brassicasterol | BRE% | % |
| campesterol free | CAF | mg/l |
| campesterol ester | CAE | mg/l |
| campesterol total | CAT | mg/l |
| esterification rate of campesterol | CAE% | % |
| cholesterol free | CHF | mg/l |
| cholesterol ester | CHE | mg/l |
| cholesterol total | CHT | mg/l |
| esterification rate of cholesterol | CHE% | % |
| lanosterol free | LAF | mg/l |
| sum of desmosterol, dymosterol, 7-dehydrocholesterol 1 free | DEZY7DHCF | mg/l |
| sum of DEZY7DHC ester | DEZY7DHCE | mg/l |
| sum of DEZY7DHC total | DEZY7DHCT | mg/l |
| esterification rate of DEZY7DHC | DEZY7DHCE% | % |
| β-sitosterol free | SIF | mg/l |
| β-sitosterol ester | SIE | mg/l |
| β-sitosterol total | SIT | mg/l |
| esterification rate of β-sitosterol | SIE% | % |
| stigmasterol free | STF | mg/l |
| stigmasterol ester | STE | mg/l |
| stigmasterol total | STT | mg/l |
| esterification rate of stigmasterol | STE% | % |
| **amino acids** |  |  |
| ratio branched chain/aromatic amino acids ratio | Fisher's ratio |  |
| alanine | Ala | μmol/l |
| aminobutyric acid | Aba | μmol/l |
| arginine | Arg | μmol/l |
| aspartic acid | Asp | μmol/l |
| carnosine | Carnosin | μmol/l |
| citrulline | Cit | μmol/l |
| glutamine | Glut | μmol/l |
| glutamic acid | Glut | μmol/l |
| methyl glutamic acid | MeGlu | μmol/l |
| glycine | Gly | μmol/l |
| histidine | His | μmol/l |
| methylhistidine | MeHis | μmol/l |
| hydroxyproline | OHProl | μmol/l |
| leucine/isoleucine | Leu/Ile | μmol/l |
| lysine | Lys | μmol/l |
| methyl malonic acid | MMA | μmol/l |
| methionine | Met | μmol/l |
| ornithine | Orn | μmol/l |
| phenylalanine | Phe | μmol/l |
| pipecolic acid | PiPA | μmol/l |
| proline | Pro | μmol/l |
| sarcosine | Sarc | μmol/l |
| serine | Ser | μmol/l |
| taurine | Tau | μmol/l |
| threonine | Thr | μmol/l |
| tryptophane | Trp | μmol/l |
| tyrosine | Tyr | μmol/l |
| valine | Val | μmol/l |
| **Carnitines** |  |  |
| free carnitine | C0 | μmol/l |
| acetylcarnitine | C2 | μmol/l |
| propionylcarnitine | C3 | μmol/l |
| malonylcarnitine | C3DC | μmol/l |
| butyrylcarnitine | C4 | μmol/l |
| hydroyxbutyrylcarnitine | C4 OH | μmol/l |
| valerylcarnitine | C5 | μmol/l |
| 2-hydroxyisovalerylcarnitin | C5 OHHMG | μmol/l |
| tigylcarnitine | C5:1 | μmol/l |
| hexanoylcarnitine | C6 | μmol/l |
| methylmalonylcarnitine | C6DC | μmol/l |
| octanoylcarnitine | C8 | μmol/l |
| octenoylcarnitine | C8:1 | μmol/l |
| decanoylcarnitine | C10 | μmol/l |
| decenoylcarnitine | C10:1 | μmol/l |
| dodecanoylcarnitine | C12 | μmol/l |
| tetradecanoylcarnitine | C14 | μmol/l |
| tetradecenoylcarnitine | C14:1 | μmol/l |
| 3-hydroxy-tetradecanoylcarnitin | C14 OH | μmol/l |
| hexadecanoylcarnitine | C16 | μmol/l |
| hexadecenoylcarnitine | C16:1 | μmol/l |
| hydroxyhexadecenoylcarnitine | C16:1 OH | μmol/l |
| hydroxyhexadecanoylcarnitine | C16 OH | μmol/l |
| octadecanoylcarnitine | C18 | μmol/l |
| octadecenoylcarnitine | C18:1 | μmol/l |
| hydroxyoctadecenoylcarnitine | C18:1 OH | μmol/l |
| octadecadienylcarnitine | C18:2 | μmol/l |
| hydroxy-octadec-2-enoylcarnitin | C18:2 OH | μmol/l |
| hydroxy-octadec-1-anoylcarnitin | C18 OH | μmol/l |
| eicosaeniccarnitin | C20:1 | μmol/l |
| eicosadieniccarnitin | C20:2 | μmol/l |
| eicosatetraeniccarnitin | C20:3 | μmol/l |

**Figure legends supplemental figures**

**Fig. S1 Correlation of circulating sterols to routine parameters and the MEAF**

a Correlation of free (SIF), esterified (SIE), total (SIT), and the esterification rate of SI (SIE%) to the MEAF, ALT, bilirubin, and the INR.

b Correlation between the esterification rate of each sterol and the MEAF.

The correlation coefficient R^2^ and the level of significance (* p<0.05, ** p<0.01, *** p<0.001) at day 3, 6, and 9 post-LTX are shown.

**Fig. S2 Amino acids and acylcarnitines as predictors for a high MEAF**

Receiver operating characteristic (ROC) analysis to verify which parameter predicts high MEAF. The 75% percentile of the MEAF was used as binary classifier, i.e. the 25% of the patients with a MEAF ≥ 6.10 were compared to the others. Values for the area under the curve (AUC; 0-1) and p-values are shown in heat maps.

**Fig. S3 Amino acids and acylcarnitines as predictors for 3-month mortality**

Receiver operating characteristic (ROC) analyses to verify which parameter predicts clinical outcome. 3-month mortality was used as binary classifier. Explanations see figure S2.
