# Supplementary figures and images for: Circulating sterols as predictors of early allograft dysfunction and clinical outcome in patients undergoing liver transplantation
Source: Metabolomics. 2016 Oct 24;12(12):182. doi: 10.1007/s11306-016-1129-z (PMC5078158; doi:10.1007/s11306-016-1129-z)

**a**

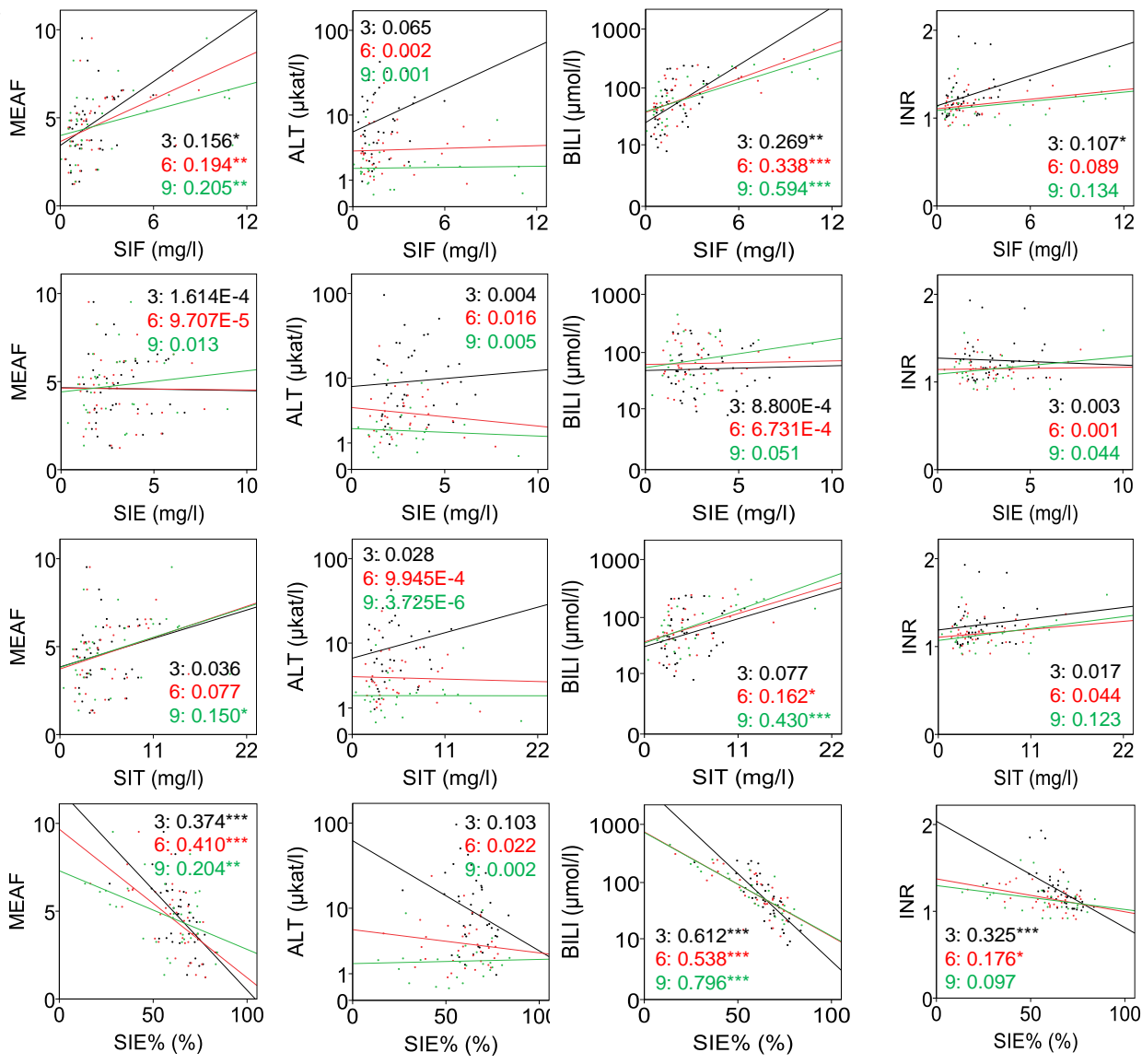

**b**

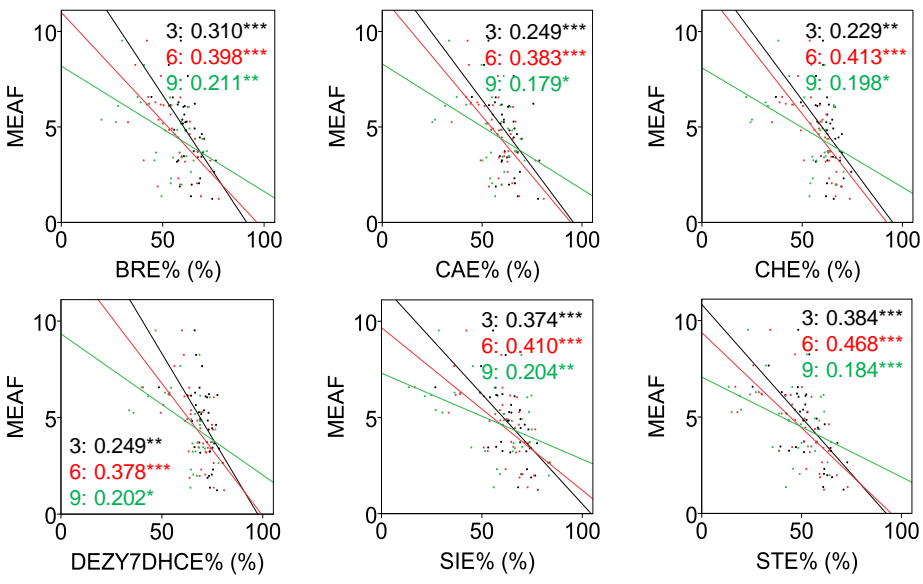

Figure S1

Supplement: Supplementary file 2 — Supplementary material 2 (PDF 361 kb) [file 11306_2016_1129_MOESM2_ESM.pdf]

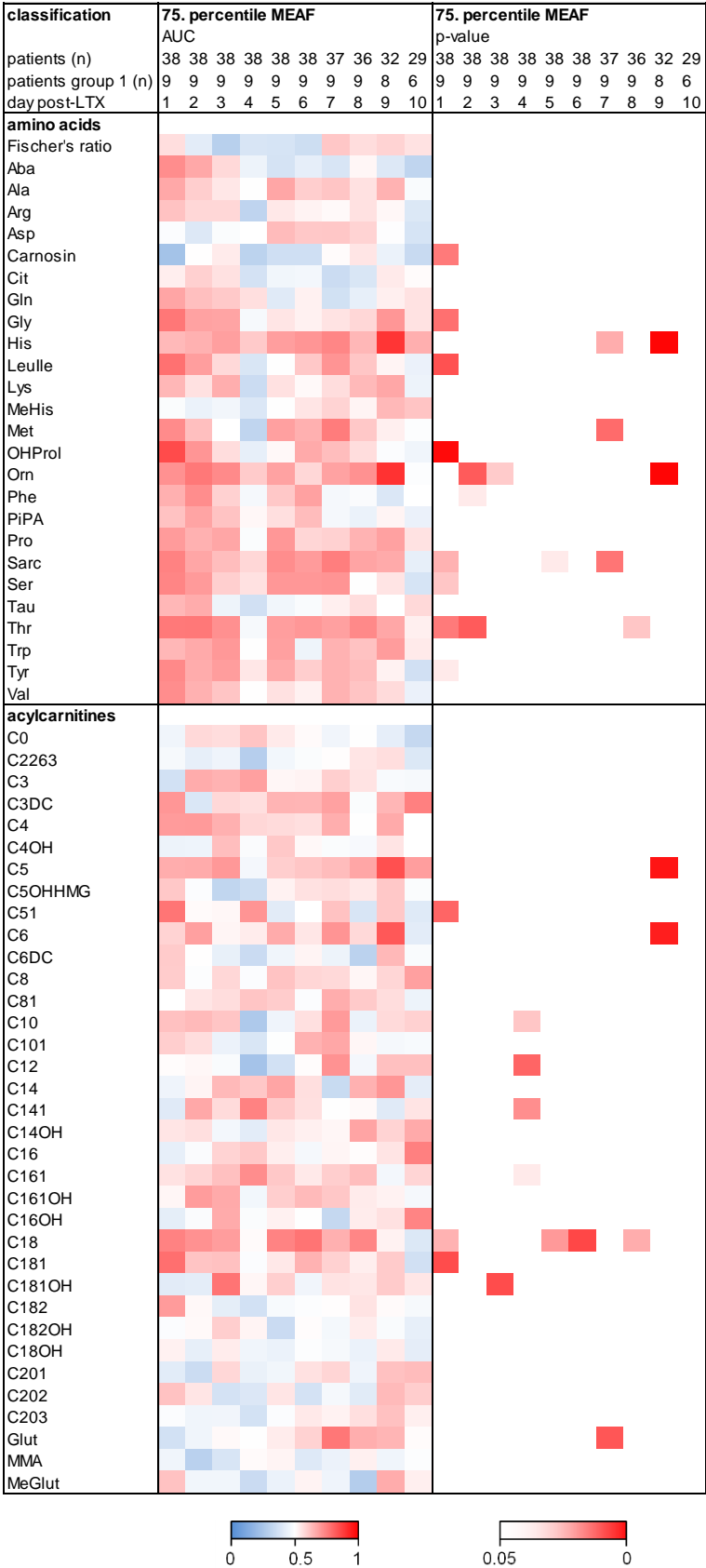

Figure S2

Supplement: Supplementary file 3 — Supplementary material 3 (PDF 15 kb) [file 11306_2016_1129_MOESM3_ESM.pdf]

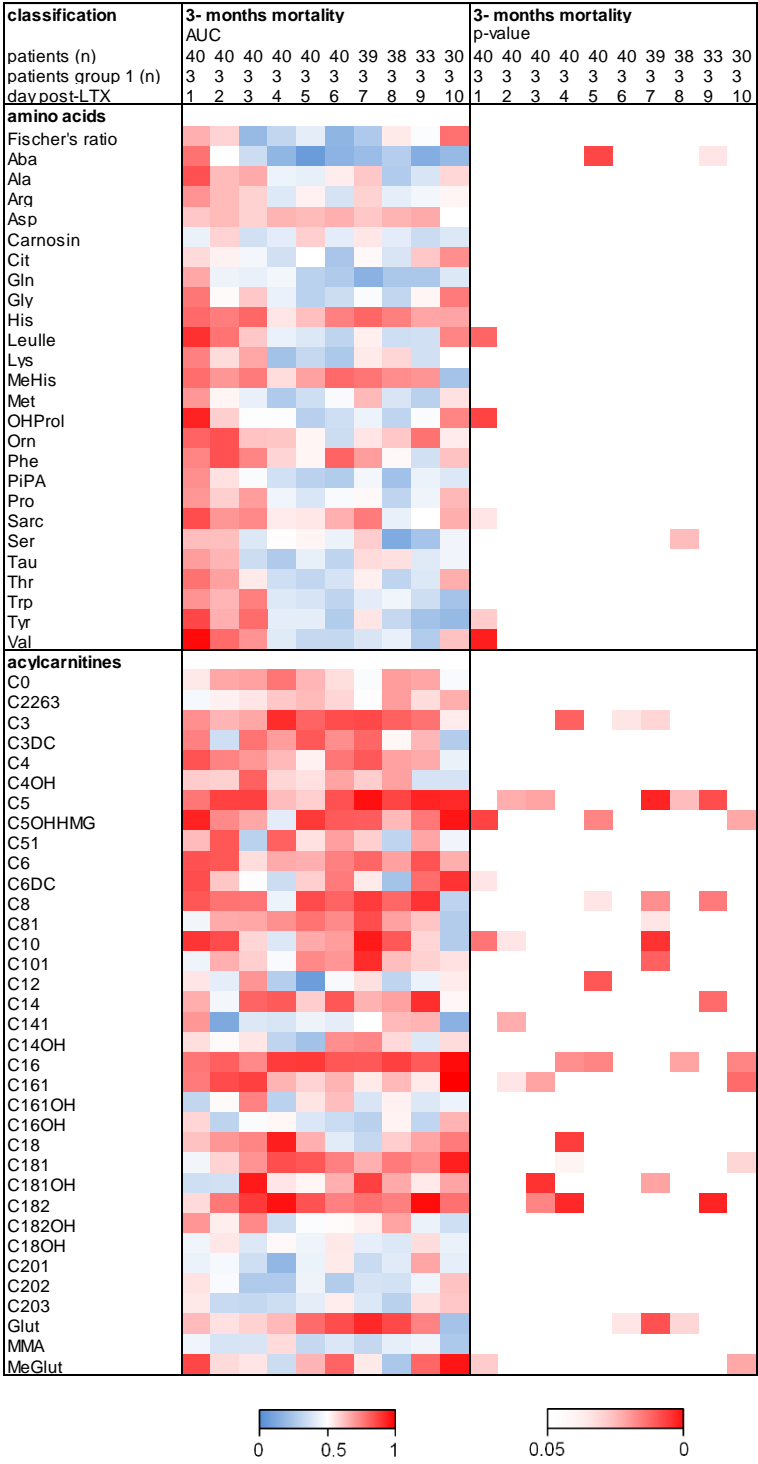

Figure S3

Supplement: Supplementary file 4 — Supplementary material 4 (PDF 15 kb) [file 11306_2016_1129_MOESM4_ESM.pdf]
